# Cumulative Childhood Trauma and Couple Satisfaction: Examining the Mediating Role of Mindfulness

**DOI:** 10.1007/s12671-020-01390-x

**Published:** 2020-05-11

**Authors:** Natacha Gobout, Francis Morissette Harvey, Gaëlle Cyr, Claude Bélanger

**Affiliations:** 1 Department of Sexology, Université du Québec à Montréal (UQAM), C.P. 8888 Succursale Centre-Ville, Montreal, Quebec H3C 3P8 Canada; 2 Université du Québec à Montréal (UQAM) and Interdisciplinary Research Center on Intimate Relationship Problems and Sexual Abuse (CRIPCAS), Montreal, Quebec Canada; 3 Department of Psychology, Université du Québec à Montréal (UQAM), Montreal, Quebec Canada

**Keywords:** Childhood cumulative trauma, Mindfulness, Acting with awareness, Non-judgement, Observing, Describing, Non-reactivity, Couple satisfaction

## Abstract

**Objectives:**

Cumulative childhood trauma (CCT) survivors are at a higher risk of suffering from interpersonal problems including couple dissatisfaction. Dispositional mindfulness is increasingly proposed as a potential explanatory mechanism of post-traumatic symptomatology and has been documented as a predictor of couple satisfaction. Most authors operationalize mindfulness as a multidimensional disposition comprised of five facets (i.e., Describing, Observing, Non-judgment of inner experiences, Non-reactivity, and Acting with awareness), but the role of these facets in the link between CCT and couple satisfaction has yet to be understood. This study aimed to assess mindfulness as a potential mediator in the relationship between CCT and couple satisfaction and to examine the distinctive contributions of mindfulness facets in this mediation.

**Methods:**

A sample of 330 participants from the community completed measures of couple satisfaction, mindfulness, and exposure to eight types of childhood maltreatment experiences.

**Results:**

Path analysis results revealed that mindfulness mediated the relationship between CCT and couple satisfaction. More precisely, two mindfulness facets acted as specific mediators, namely, Describing and Non-judgment of inner experiences. The final integrative model explained 14% (*p* < .001) of the variance in couple satisfaction.

**Conclusions:**

Findings suggest that mindfulness may be a meaningful mechanism in the link between CCT and couple satisfaction. They also highlight that description of inner experiences and a non-judgmental attitude of these experiences may act as key components to understand the influence of CCT on adults’ lower couple satisfaction.

The endemic nature of childhood trauma has been shown in an increasingly larger body of research. Indeed, the landmark epidemiological studies led by Finkelhor et al. (2007, 2009) revealed that the majority of adults from the general population reported having experienced at least one type of trauma during childhood, while 22% reported having sustained four types of trauma or more. These results along with those of other researchers highlight a phenomenon described as cumulative childhood trauma (CCT; Cloitre et al. 2009; Hodges et al. 2013), which refers to the accumulation of several types of interpersonal trauma sustained during childhood such as physical, psychological, or sexual maltreatment. The high rates of childhood revictimization and co-occurrence between different types of trauma are particularly alarming, since CCT is associated with a more severe and complex symptomatology than single traumatic experience (Bigras et al. 2017; Briere et al. 2010). CCT takes place within an interpersonal context; it is committed by a human being and typically within a significant relationship. Due to the human bond between CCT survivors and perpetrators, CCT may be related to interpersonal difficulties in general, and may particularly impair couple satisfaction (Godbout et al. 2014). In addition, establishing and nurturing a satisfactory intimate relationship represent one of the main goals of adult interpersonal life and is amongst the strongest predictors of adult physical and psychological health and well-being (Holt-Lunstad et al. 2010; Vaillant & Mukamal 2001). For these reasons, understanding the impact of CCT on couple satisfaction and the variables explaining this link is essential.

The early developmental stage of CCT survivors, as well as their position of dependency and vulnerability to their abuser, makes these traumatic experiences particularly overwhelming and detrimental to long-term physical and psychological wellness (Masten & Wright 1998; McCrory & Viding 2015). CCT is associated with a vast array of long-lasting repercussions, including post-traumatic stress, self-regulatory problems, depression, and anxiety symptoms (Dugal et al. 2016; Norman et al. 2012; Suliman et al. 2009). Moreover, CCT survivors tend to grow up with feelings of mistrust and fear of being abandoned, which may be projected into their adult relationships, specifically within their couple relationship (Bowlby 1969; Godbout et al. 2017b). The intimate relational context of a romantic relationship typically carries many physical and psychological stimuli that might bring back dysfunctional and painful interpersonal representations (Briere 2002).

In that respect, a study by Colman and Widom (2004) revealed that adult interpersonal trauma survivors were twice as likely as non-survivors to get divorced. Several studies also observed that survivors tend to avoid intimate relationships, reporting that they fear being hurt or revictimized (Rumstein-McKean & Hunsley 2001; Staples et al. 2012), and that they are at higher risks of couple distress as well as intimate partner violence (Bigras et al. 2015; Godbout et al. 2007; Nguyen et al. 2017). Significant links were also found between post-traumatic avoidance behaviors and relational commitment difficulties (Staples et al. 2012). Yet, even though the association between interpersonal trauma and couple satisfaction is increasingly documented, empirical literature on the matter reveals inconsistent findings, such as weak or absent direct link (e.g., Godbout et al. 2006; Whisman 2006). This suggests possible intermediary variables, but the explanatory mechanisms of this relation remain sparsely understood. Mindfulness might shed light on this phenomenon.

The concept of mindfulness has sparked a growing interest in the field of psychology and mental health. Kabat-Zinn (2003) described mindfulness as the ability to pay attention to the present moment in a purposeful and non-judgmental manner. Although mindfulness may be conceptualized as a temporary mental state or as a form of wellness practice such as mindfulness meditation, most authors conceptualize it as a stable, multifaceted, and individual disposition (e.g., Blanke & Brose 2017; Dekeyser et al. 2008). Based on that definition, lower dispositional mindfulness levels may partially explain the link between CCT and adults’ current couple satisfaction. For example, the avoidance of painful internal experiences through dissociative states or tension-reducing behaviors such as substance abuse and self-harm represents the antithesis of mindful behavior. This strategy might become persistent and may be maintained by conditioning processes including negative reinforcement. These overdeveloped avoidance responses to stress and adversity potentially prevent trauma-related effects from being psychologically processed, thus hindering survivors’ psycho-relational well-being, including couple satisfaction. This entrapping cycle of avoidance and suffering has been presented as a *pain paradox* (Briere 2015*)* or *behavioral loop* (Hayes & Gifford 1997) and offers a theoretical ground to the potential role of mindfulness in the link uniting both CCT and couple satisfaction. Recent studies supported this theoretical standpoint by revealing a link between CCT and lower dispositional mindfulness and between lower dispositional mindfulness and increased psychological distress and psychosocial impairment (Bolduc et al. 2018; Kratzer et al. 2018; Nitzan-Assayag et al. 2015). In his literature review, Kozlowski (2013) highlighted a large body of empirical studies showing direct and indirect associations between dispositional mindfulness and couple satisfaction. Moreover, in their mixed-methods randomized clinical trial of a mindfulness-based relationship education program, Gambrel and Piercy (2015a, 2015b) found significant improvement with couple satisfaction in men, while both men and women reported experiencing better couple satisfaction in their study’s qualitative portion, also providing support to the postulate that increased dispositional mindfulness might be related to higher couple satisfaction.

Based on previous validated measures, Baer et al. (2006) proposed an integrative mindfulness model that is composed of five facets: (1) observing, which is defined as the ability to notice one’s inner experiences, including thoughts and feelings, as they unfold, (2) non-judgment, defined as the ability to accept one’s inner experiences without judgment, (3) describing, defined as the ability to label one’s inner experiences, (4) non-reactivity, defined as the ability to allow one’s inner experiences to come and go without getting caught up in them, and (5) acting with awareness, defined as the ability to focus one’s attention on what is happening in the here and now. These five facets are widely used in empirical literature to discriminate which inherent aspects of dispositional mindfulness are related to various mental health conditions as well as non-pathological individual differences. Besides enabling a better construct validity (Baer et al. 2006; Blanke & Brose 2017; Park et al. 2013), this multifaceted operationalization of mindfulness allows the examination of the potential distinctive role of mindfulness facets in regard to the link uniting CCT to couple satisfaction. A non-judgmental, accepting attitude towards inner experiences was identified as especially relevant to post-traumatic adaptation (Boughner et al. 2016; Thompson & Waltz 2010; Vujanovic et al. 2009), and might also be generalized to the partner, fostering empathetic and pro-relationship behaviors (Barnes et al. 2007; Birnie et al. 2010), hence potentially acting as a mediator between CCT and couple satisfaction. Several authors (e.g., Pepping & Halford 2016; for a review see Karremans et al. 2017) suggested that being more connected to one’s own affects might improve couple interactions through enhanced emotion identification, regulation, and communication. Observing and describing might therefore also play a significant role in the link between CCT and couple satisfaction. Furthermore, reduced mindful awareness was found in CCT survivors (Bolduc et al. 2018). However, it was suggested that mindful awareness might reduce the occurrence of mindless acts towards the romantic partner (Papies et al. 2015) and may foster conflict management skills (Johns et al. 2015; Shapiro et al. 2008), hence a lower disposition to acting with awareness in the aftermaths of CCT might potentially be related to a decreased couple satisfaction. However, the postulated mediator roles of the mindfulness facets in the link uniting CCT and couple satisfaction remain speculative and need to be empirically examined within an integrative model. Information on the links between CCT, couple satisfaction, and mindfulness could improve our understanding of trauma-related symptomatology and lay the groundwork for the development of better services for trauma survivors and couples.

The present study had two main objectives: the first objective was to examine the mediating role of mindfulness in the link between CCT and couple satisfaction and the second objective was an exploratory examination of the role of each of the five mindfulness facets in this relation. Based on previous findings, it was hypothesized that higher CCT would be related to lower relational satisfaction, through lower mindfulness. It was also hypothesized that each of the mindfulness facets would play distinct roles in this association. More precisely, it was hypothesized that CCT would be related to lower observing, non-judgment, describing, and acting with awareness, which in turn would lead to decreased couple satisfaction. Due to insufficient data in previous studies, no precise hypothesis was postulated regarding non-reactivity.

## Method

### Participants

The total sample consisted of 330 participants (232 women and 98 men), who were in a romantic relationship. Participants were either married (16.7%, *n* = 55) in a common-law union (48.8%, *n* = 161) or in a long-term relationship with a non-cohabiting partner (34.5%, *n* = 114). Participants’ mean age was 29.9 years (*SD* = 10.43). Most participants spoke French as their first language (90.6%, *n* = 298) and identified as heterosexual (91.7%, *n* = 300). Amongst participants, 49.1% were students (*n* = 159), 38.3% were full-time workers (*n* = 124), and 11.4% (*n* = 37) were part-time workers. Most of them were Canadians (94.5%, *n* = 312) and were university educated (71.5%, *n* = 236). Almost half of the sample (44.4%, *n* = 146) reported an annual income below $19,000 CAD, which would classify them under the Quebec province low-income threshold (Statistique Canada, 2020). Results of an a priori Monte Carlo analysis performed on Mplus (Muthén & Muthén 2017) indicated that 330 participants were sufficient to detect weak-to-moderate associations in the hypothesized integrative model, with a standard type I error rate (*α* = .05), and a power of .80.

### Procedure

Participants from the general population were recruited on a voluntary basis using social media and diffusion lists, including a Facebook page dedicated to the research project, Twitter, and general lists for research in psychology. The study was introduced as an exploration of the effect of childhood traumatic experiences on adult intimate relationships. A hyperlink led participants to SurveyMonkey (www.surveymonkey.com), an anonymous and secure online survey platform. This study was approved by the Institutional Review Board for research involving human subjects of the *Université du Québec à Montréal*.

### Measures

#### Cumulative Childhood Trauma

CCT was assessed using the Cumulative Childhood Trauma Questionnaire (CCTQ; Godbout et al. 2017a). The CCT evaluates childhood sexual abuse, as defined by Canadian law, referring to any sexual act between a child under 16 years of age and a person who is five or more years older, or who is in a position of authority or any unwanted sexual act experienced prior to age 18. The CCT also measures seven additional types of trauma using a 7-point Likert scales: physical and psychological abuse, psychological and physical neglect, witnessed physical and psychological violence between parental figures, and peer bullying. To compute a total score, each trauma type is coded as having been experienced at least once in a typical year before the age of 18 (1) or not (0), and the total CCT score is obtained by summing the participants’ dichotomous scores on the eight types of trauma. Total CCT score can range 0–8, with higher scores indicating greater CCT exposure. The use of this composite CCT variable is recommended in trauma-focused empirical literature (e.g., Bigras et al. 2017; Hodges et al. 2013), it represents a composite variable of cumulative risk (see Appleyard et al. 2005) that is statistically sensitive even with small samples, which makes no assumptions about the relative strength of multiple risk factors, and avoid measurement error associated with the analysis of highly correlated variables (Evans et al. 2013). In the present sample, the alpha coefficient was satisfactory (*α* = .71).

#### Couple Satisfaction

Couple satisfaction was measured using the 4-item French version of the Dyadic Adjustment Scale (DAS-4; Sabourin et al. 2005). The DAS (Spanier 1976) is the most widely used measure to assess couple satisfaction in both clinical and non-clinical populations (Graham et al. 2006; Whisman et al. 2018). Participants reported the degree to which each item describes their romantic relationship during the past month on 5- or 6-point Likert scales. Total scores ranged between 0 and 21, with higher scores indicating higher couple satisfaction, and a cutoff score of 13 to distinguish distressed individuals from those who are relatively satisfied with their relationship. The psychometric properties of the DAS-4 are well established (Bigras et al. 2015; Sabourin et al. 2005). In the present sample, Cronbach’s alpha coefficient was good (*α* = .80).

#### Mindfulness

Mindfulness was assessed using the French version of the Five Facet Mindfulness Questionnaire (FFMQ; Baer et al. 2006; Heeren et al. 2011). The FFMQ is a 24-item self-reported questionnaire on a 5-point Likert scales, measuring mindfulness with five subscales: Describing, Observing, Non-judgment, Non-reactivity, and Acting with awareness. The FFMQ is one of the most commonly used measures to assess mindfulness and has been validated with both clinical and non-clinical populations (Aguado et al. 2015; Veehof et al. 2011). The French version of the FFMQ presents good psychometric properties (Heeren et al. 2011), and a confirmatory factor analysis supported the five-factor structure of the construct in several populations (e.g., Bohlmeijer et al. 2011; Gu et al. 2016). In the present sample, Cronbach’s alpha was satisfactory (*α* = .77).

### Data Analyses

Descriptive analyses and Pearson’s correlations were generated using IBM SPSS version 22. Two-tailed independent sample *t* tests were performed to examine potential gender differences, and chi-square tests were performed for potential differences in the frequencies of endured trauma types across genders, using the phi coefficient (*ϕ*) as the effect size indicator. Multivariate outliers were investigated using Mahalanobis distance at a criterion of *p* < .001, which did not detect any anomalies (Aggarwal 2017). Path analyses were then conducted using Mplus (Muthén & Muthén 2017), which is robust to non-normality and accounts for missing data through the use of maximum likelihood estimation with robust standard errors (MLR) to test the hypothesized mediation models. The first model used the global mindfulness score as a mediator to test the general mediation role of mindfulness, whereas the second model used the five FFMQ subscales to examine the specific mediating roles of each mindfulness facet while controlling for their shared effect. A 10,000 resampling bias-corrected bootstrap with a 95% confidence interval was used to examine the indirect effects, which is considered significant if the generated interval does not include zero (Preacher & Hayes 2008). The fit of the estimated model to the observed data was assessed using several adjustment indices: the comparative fit index (CFI), the root mean square error of approximation (RMSEA), and the chi-square value (*χ*^2^). CFI values equal or superior to .95, and a RMSEA value inferior to .08, are considered indicators of good fit (Hooper et al. 2008; Hu & Bentler 1999). A non-statistically significant *χ*^2^ value indicates a good fit but is sensitive to large sample sizes, so the *χ*^2^/*df* ratio was also used in the estimation, where a value inferior to 3 indicates a good fit (Kline 2015).

To verify if the model was a good representation of the patterns amongst the data for both men and women, we also tested the gender invariance of the model. First, adequacy of model fit for the configural model was assessed across men and women, letting all structural paths vary freely. We then assessed the fit of the structural model across men and women after adding equality constraints on each path. Finally, the configural and structural models’ fits were compared using a chi-square test, where a significant univariate incremental chi-square value (*p* < .05) indicates differences between men and women. In the case of a significant difference, a series of Wald tests in which all structural path, except one, were constrained to be equal across genders that was performed in order to identify the explanatory variables of the gender differences.

## Results

### Descriptive Data

Descriptive analysis revealed that 87.3% (*n* = 288) of participants reported at least one type of childhood interpersonal trauma, and that the mean number of trauma types was 3.09 for the total sample. This is similar to results from other studies on samples from the general population (e.g., Bigras et al. 2017; Briere et al. 2010). Frequencies of the different interpersonal trauma types, across genders and for the total sample, are presented in Table 1. Clinical levels of couple dissatisfaction were reported by 11.5% (*n* = 38) of participants.

**Table 1:** Prevalence of childhood interpersonal traumas across gender

	Women		Men		Total sample	
	*n*	%	*n*	%	*n*	%
Sexual abuse	62	26.8%	25	25.5%	87	26.4%
Physical violence	75	32.5%	52	53.1%	127	38.6%
Psychological violence	104	45%	43	43.9%	147	44.7%
Physical neglect	19	8.2%	20	20.4%	39	11.9%
Psychological neglect	147	63.6%	62	63.3%	209	63.5%
Interparental physical violence	24	10.4%	15	15.3%	39	11.9%
Interparental psychological violence	122	52.8%	54	55.1%	176	53.5%
Bullying	122	52.8%	72	73.5%	194	59%

One participant was excluded from comparisons across gender due to missing data (*N* = 329)

Table 2 shows Pearson’s correlations between variables of interests across genders, as well as the means and standard deviations. The bivariate correlational analyses revealed that, for both genders, mindfulness was positively correlated to couple satisfaction and negatively correlated to CCT. In addition, CCT was negatively correlated to couple satisfaction in the total sample and amongst women (*r* [328] = − .18, *p* < .01), but not amongst men (*r* [328] = − .16, *p =* .11).

**Table 2:** Means, standard deviations, and correlations Amongst study variables

	1.	2.	3.	4.	5.	6.	7.	8.	*M*	*SD*
1. CCT	–								3.09	2.08
2. Couple Satisfaction	− .170**	–							16.59	3.32
3. Mindfulness	− .242**	.322**	–						82.53	11.71
4. Describing	− .202**	.305**	.759**	–					18.13	4.13
5. Non-Reactivity	− .038	.038	.499**	.289**	–				14.24	3.69
6. Non-Judgment	− .203**	.229**	.459**	.198**	−.116*	–			16.92	3.99
7. Awareness	− .295**	.195**	.677**	.440**	.013	.449**	–		19.47	4.03
8. Observing	.027	.160**	.519**	.270*	.324**	−.183**	.062	–	13.79	4.22

*CCT* childhood cumulative trauma, *mindfulness* total FFMQ score, *awareness* acting with awareness

*p < .05, **p < .01, ***p < .001

Results from the *t* tests showed that men (*M* = 81.89, *SD* = 11.35) had lower mindfulness levels than women (*M* = 82.85, *SD* = 11.87; *t* [327] = − 684, *p* < .05) and higher levels of CCT (*M* = 3.5, *SD* = 2.18) than women (*M* = 2.92, *SD* = 2.01; *t* (327) = − 2.32, *p* < .05). No significant gender differences were observed for the couple satisfaction scale (men: *M* = 16.58, *SD* = 3.26; women: *M* = 16.58, *SD* = 3.35, *t* [327] = − .01, *p* = .99). Regarding the frequency of endured trauma across genders, chi-square tests indicated that a higher proportion of women reported having experienced peer bullying as compared to men, *χ*^2^ (1) = 12.13, *p* < .001, *ϕ* = .192. More women than men reported having sustained physical violence by caregivers, *χ*^2^ (1) = 12.31, *p* < .001, *ϕ* = .193. However, a higher number of men reported physical neglect when compared with women, *χ*^2^ (1) = 9.77, *p* < .01, *ϕ* = .172.

### Mediation Model

**Fig. 1:**
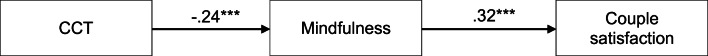
Path analysis model of the relation between childhood cumulative trauma, mindfulness, and couple satisfaction. CCT, childhood cumulative trauma; mindfulness, total FFMQ score. *p < .05, **p < .01, ***p < .001

First, a path analysis was performed and revealed a significant association between CCT and lower couple satisfaction (*β* = − .17, *p* < .01), which explained 3% of the variance in the outcome. Second, the mediator was added to the model; this significant direct effect became non-significant when the global mindfulness score was introduced as a mediator in the model (see Fig. 1). Results showed that higher CCT predicted lower mindfulness (*β =* − .24, *p* < .001), which in return predicted lower levels of couple satisfaction (*β =* .32, *p* < .001). The bootstrap procedure confirmed a significant indirect effect (*β* = .03, 95% CI [− .059, − .016]), supporting the mediation hypothesis. The fit indicators revealed an adequate adjustment between the data and the hypothesized mediation model, *χ*^2^ (1) = 3.19, *p* = .07, *χ*^2^/*df* = 3.19, CFI = .955, TLI = .866, RMSEA = .081, 95% CI [.000, .189]. This model explained 10% of the variance for couple satisfaction.

### Integrative Mediational Model Across the Mindfulness Facets

**Fig. 2:**
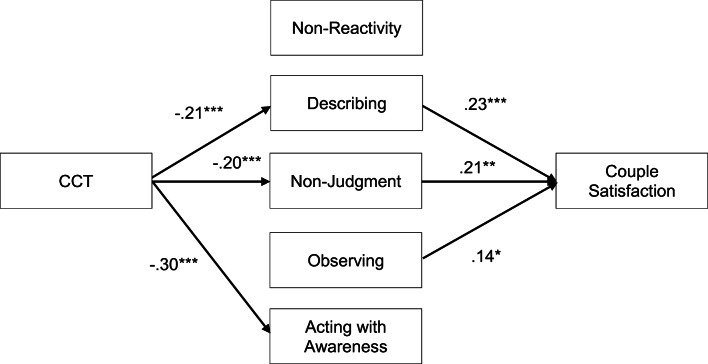
Integrative model for the mediating role of the mindfulness facets in the relationship between childhood cumulative trauma and couple satisfaction. CCT, childhood cumulative trauma. *p < .05, **p < .01, ***p < .001

The direct link between CCT and couple satisfaction also became non-significant after introducing the mediators in the models. Results of path analysis confirmed that CCT was associated to lower scores on the Describing and Non-judgment mindfulness scales, which, in return, both predicted lower couple satisfaction scores (see Fig. 2). The Non-reactivity scale was not related to CCT nor to couple satisfaction. Therefore, this variable was withdrawn from the model for parsimony. The bootstrap procedure revealed two significant indirect effects of CCT on couple satisfaction, through Describing (*β =* − .02, 95% CI [− .040, − .008]) and Non-judgment (*β = −*.02, 95% CI [−.038, −.006]), hence, confirming two mediation effects in the model. Residual covariances were estimated between the following facets in the model: Observing and Describing (*r* [328] = .26, *p* < .001), Observing and Non-judgment (*r* [328] = − .21, *p* < .001), Describing and Acting with awareness (*r* [328] = .39, *p* < .001), Describing and Non-judgment (*r* [328] = .16, *p* < .01), and Acting with awareness and Non-judgment (*r* [328] = .43, *p* < .001). The adjustment indicators showed satisfying fit, *χ*^2^ (4) = 5.02, *p* = .29, *χ*^2^/*df* = 1.26, CFI = .996, TLI = .985, RMSEA = .028, CI [.000, .092]. The global model explained 14% of the variance of couple satisfaction.

### Gender Invariance

The adjustment indicators for the configural model showed a satisfactory representation of the data across women and men, *χ*^2^/*df* = .774, CFI = 1.000 TLI = 1.026, RMSEA = .000, CI [.000; .077]. This model was then compared to the structural model, where the structural paths were constrained to be equal across genders, using a chi-square test. The results revealed a significant difference between women and men, *χ*^2^ (11) = 19.95, *p <* .05. The Wald test procedure revealed a marginally significant difference across genders (*p* = .05) in the link between the describing scale and couple satisfaction (men: *β* = .35, *p* < .001; women: *β* = .19, *p* = .01), which imply that the relation between the describing facet and couple satisfaction was stronger for men compared with women. A second chi-square test was then performed, which indicated no significant differences between genders once the constraint on this path was removed, *χ*^2^(10) = 17.15, *p* = .07.

## Discussion

The goal of this study was to determine whether dispositional mindfulness mediated the relationship between CCT and couple satisfaction. Results confirmed the hypothesized mediation model and showed that CCT is associated with lower levels of mindfulness, which in turn are related with lower couple satisfaction. These results are in line with past studies on child maltreatment, which showed detrimental effects of CCT exposure on dispositional mindfulness (e.g., Kelly & Garland 2016; Thompson & Waltz, 2010). These findings also add up to the literature highlighting a relation between mindfulness and couple satisfaction (Kappen et al. 2018; Kozlowski 2013). Yet, the cumulative effect of exposure to several types of trauma was not considered in previous research, which is an important contribution of the current study. Results indicate that CCT is related to an impeded intimate relationship through a lower disposition to mindfulness. Authors suggested that lower mindfulness dispositions in CCT survivors may lead to disrupted intimate relationships because of a detachment or disengagement from their own and their partner’s internal states, as well as an impaired ability to communicate and lack of intimacy (Déziel et al. 2017). These results substantiate the theoretical framework guiding this study, namely, the pain paradox (Briere 2015), suggesting that mindfulness-related capacities might be beneficial to CCT survivors in enhancing their ability to make sense of their post-traumatic schemas and memories or to become better adapted to them. Conversely, avoidance-based coping strategies adopted by certain CCT survivors might tend to decrease CCT survivors’ capacity to be emotionally and intimately available to their romantic partners. Indeed, survivors’ blindness to interpersonal cues, unresolved post-traumatic schemas, and lack of awareness of their own actions might lead to major difficulties of sharing long-lasting bonds with another person (Staples et al. 2012). Since couple distress is a well-documented consequence of interpersonal trauma (Godbout et al. 2014) and one of the main reasons for consultation mentioned by patients in individual psychotherapy (Whisman & Uebelacker 2006), professionals are likely to encounter patients suffering from these issues and encouraged to understand the links between those variables.

Results also supported our second hypothesis, showing that the mindfulness facets play distinct roles in the association between CCT and couple satisfaction. More precisely, results imply that the abilities to describe and accept one’s own experience without judgment may be the active ingredients in the role of mindfulness as a mediator of the relationship between CCT and couple satisfaction. These findings are consistent with other studies (Thompson & Waltz 2010; Vujanovic et al. 2009) suggesting that a non-judging attitude towards one’s own thoughts, and affects might lessen the impacts of childhood interpersonal trauma. Our results also suggest that CCT survivors suffering from difficulties in identifying their inner experiences might be prone to couple difficulties. These findings thus imply that being able to accept and describe emotions and thoughts could be particularly central to survivors’ couple satisfaction. These results provide preliminary evidence suggesting that the capacity to label internal experiences—including feelings, thoughts, and bodily sensations—with words, within a non-judgmental mindset, may foster satisfactory couple relationship. It is likely that these capacities are especially relevant since they facilitate communication of their internal experiences to their partners, and thus promote the development of a deeper connection and intimacy. This interpretation of our results is further supported by studies highlighting the positive impact of mindfulness on communication skills (Jones & Hansen 2015), and the importance of communicational abilities in couple satisfaction (Busby et al. 2001; Tavakolizadeh et al. 2015).

The roles of mindfulness facets need to be interpreted while taking into account the correlations between the subscales, observed both in this study and in previous research (Baer et al. 2006). Thus, although the survivor’s lower tendency towards objective consideration of their thoughts and feelings (Non-judgment), combined with their lower capacity to recognize and label them (Describing), most directly explain the mediation role of mindfulness in our models, the other correlated facets of mindfulness are likely to play a holistic role in the lower relational capacity of survivors. For example, the impaired survivors’ ability to stay present and aware in the present moment (Acting with awareness) might directly impede their capacities to observe and describe without judgment, which in turn directly relate to couple satisfaction. Our integrative model also depicted a significant link between Observing and couple satisfaction. This ability to observe immediate experience may potentially promote couple satisfaction by adding clarity to the current experience, not only for the respondent but also for his/her partner, offering a strong foundation in which to build a satisfying intimate interpersonal relationship through attention deployment. Another hypothesis might be that observing skills may allow the person to take notice of the nature and effects of his/her internal states and behaviors, in order to eventually discuss them with the partner, potentially using describing skills, and address problematic behaviors. Yet, previous studies have found unstable or unexpected relationships in regard to the observing dimension of mindfulness, including a weak or non-significant link with psychological well-being (e.g., neuroticism, anxiety; Baer et al., 2006; Fisak & Von Lehe 2012; Iani et al. 2017). The current findings underlie, for the first time, the relationships between specific dimensions of mindfulness and relational well-being. More precisely, they provide initial evidence regarding the role of “How” (i.e., non-judgment) and “What” (i.e., describing and observing) skills of mindfulness (Baer et al. 2006; Linehan 2014) as key components fostering couple satisfaction.

Although the observation of the experience as it unfolds and a non-judgmental attitude were both independently related to higher couple satisfaction, they were found to be negatively interrelated. Previous authors found similar results. In their study on the FFMQ patterns in well-experienced meditators, Lilja et al. (2013) found a cluster of participants showing high score on Observing, along with low scores on Non-judgment. This result mirrors the negative association between those two subscales of mindfulness observed in the current study on participants from the community. A possible explanation to this association might be that observing is related to a tendency to label the observed internal states or behaviors as inappropriate, leading to increased judgment. However, this postulate remains speculative and would need to be confirmed empirically by future research.

The absence of links between the Non-reactivity facet and both CCT and couple satisfaction is unexpected, since this scale filled a prominent role in other studies relating to childhood trauma survivors (e.g., Elices et al. 2015). Yet again, the propensity to remain calm and objective when faced with thoughts or strong feelings might be crucial for the deployment of the other facets of mindfulness (i.e., Acting with awareness, Observing, and Non-judgment). As such, the different facets of mindfulness might interact in useful manners, and the current findings may be partially explained by the correlations and complex interactions between them. More research is needed to further understand the specific role of Non-reactivity to inner experiences in the relation uniting CCT and couple satisfaction.

Lastly, the results of the gender invariance analyses indicate a good representation of the data by the hypothesized mediation model in both men and women, with partial invariance. The specific variation between gender lied in the structural path uniting men and women’s capacity to recognize and label their thoughts and feelings with words (describing) and their couple satisfaction. Findings suggest a higher propensity for men to report higher couple satisfaction when they report high tendency to identify and describe their inner experiences. Previous studies have documented gender differences in couple satisfaction predictors (e.g., Faulkner et al. 2005) and outcomes of mindfulness, most notably Gambrel and Piercy’s (2015a) clinical study, which yielded significant treatment effects solely on the male partners of their mindfulness program for couples. While more data is needed to confirm the findings, our results suggest that working on emotion, and thought description, in clinical settings might be relevant to both partners, but particularly to male trauma survivors.

### Limitations and Future Research

Interpretation of the present findings should consider the limitations of the present study. The results presented here were based on a cross-sectional methodology and cannot be considered proof of causality. In this regard, although potentially helpful in testing the fit of specific hypotheses to the actual data, path analyses cannot establish cause and effect in the absence of longitudinal data or experimental design. The proposed mediational model was based on theoretical grounds and on the established temporal sequence; CCT occurring during childhood, before current dispositional mindfulness and couple relationship. For example, the association between mindfulness and couple satisfaction could be bidirectional. Lower mindfulness might potentially lead to couple distress, which might further lower mindfulness disposition and so forth; however, only further multi-wave longitudinal studies could empirically disentangle the direction of causality amongst these variables. The study used self-reported questionnaires and may therefore suffer from common method biases including spurious correlations due to the measurement instruments, response styles, social desirability, or priming effects. The current results should be confirmed by multitraits–multimethods studies (see, Bagozzi & Yi 1993). Furthermore, participant’s ethnic identity was not reported in the questionnaire, and thus could be not considered as a covariate, which represents a limit of the actual research design. Moreover, the concept of mindfulness itself has been criticized for its lack of consensual definition and ambiguous factorial structure (see Van Dam et al. 2018). The results of this study must be appreciated while taking into account that further research, including other variables of interest, is needed to further dismantle the link between CCT, mindfulness, and couple satisfaction. Moreover, although the recruitment targeted all adults from the general population of a large metropolitan region, the sample was relatively homogenous including mostly French-Canadian, university-educated women, and further research should aim to recruit larger and more diverse samples to confirm the generalization of the results to a larger population or to a population of individuals reporting lower couple satisfaction. Moreover, the transparency of the study recruitment advertisement on childhood traumatic experiences and adult intimate relationships could potentially have introduced a self-selection bias or a bias of the responses to the questionnaires that could not have been measured with certainty. Lastly, dyadic analyses are needed in future studies to fully understand the dynamic interplay in the determinants of couple satisfaction, taking into account the interdependence between CCT experiences and mindfulness dispositions amongst the two partners.

## References

[CR1] Aggarwal, C. C. (2017). *Outlier analysis.* Springer International Publishing.

[CR2] Aguado J, Luciano JV, Cebolla A, Serrano-Blanco A, Soler J, García-Campayo J (2015). Bifactor analysis and construct validity of the five facet mindfulness questionnaire (FFMQ) in non-clinical Spanish samples. Frontiers in Psychology.

[CR3] Appleyard K, Egeland B, van Dulmen MHM, Alan Sroufe L (2005). When more is not better: The role of cumulative risk in child behavior outcomes. Journal of Child Psychology and Psychiatry.

[CR4] Baer RA, Smith GT, Hopkins J, Krietemeyer J, Toney L (2006). Using self-report assessment methods to explore facets of mindfulness. Assessment.

[CR5] Bagozzi RP, Yi Y (1993). Multitrait–multimethod matrices in consumer research: Critique and new developments. Journal of Consumer Psychology.

[CR6] Barnes S, Brown Kirk W, Krusemark E, Campbell WK, Rogge Ronald D (2007). The role of mindfulness in romantic relationship satisfaction and responses to relationship stress. Journal of Marital and Family Therapy.

[CR7] Bigras N, Daspe M-È, Godbout N, Briere J, Sabourin S (2017). Cumulative childhood trauma and adult sexual satisfaction: Mediation by affect dysregulation and sexual anxiety in men and women. Journal of Sex & Marital Therapy.

[CR8] Bigras N, Godbout N, Hébert M, Runtz M, Daspe M-È (2015). Identity and relatedness as mediators between child emotional abuse and adult couple adjustment in women. Child Abuse & Neglect.

[CR9] Birnie K, Speca M, Carlson LE (2010). Exploring self-compassion and empathy in the context of mindfulness-based stress reduction (MBSR). Stress and Health.

[CR10] Blanke ES, Brose A (2017). Mindfulness in daily life: A multidimensional approach. Mindfulness.

[CR11] Bohlmeijer E, Ten Klooster PM, Fledderus M, Veehof M, Baer R (2011). Psychometric properties of the five facet mindfulness questionnaire in depressed adults and development of a short form. Assessment.

[CR12] Bolduc R, Bigras N, Daspe M-È, Hébert M, Godbout N (2018). Childhood cumulative trauma and depressive symptoms in adulthood: The role of mindfulness and dissociation. Mindfulness.

[CR13] Boughner E, Thornley E, Kharlas D, Frewen P (2016). Mindfulness-related traits partially mediate the association between lifetime and childhood trauma exposure and PTSD and dissociative symptoms in a community sample assessed online. Mindfulness.

[CR14] Bowlby, J. (1969). *Attachment and loss*, Vol. 1: Attachment. Basic Books.

[CR15] Briere, J. (2002). Treating adult survivors of severe childhood abuse and neglect: Further development of an integrative model. In J. E. B. Myers, L. Berliner, J. Briere, C. T. Hendrix, C. Jenny, & T. A. Reid (Eds.), *The APSAC handbook on child maltreatment* (pp. 175–203). Sage.

[CR16] Briere, J. (2015). Pain and suffering: A synthesis of Buddhist and western approaches to trauma. In V. M. Follette, J. Briere, D. Rozelle, J. W. Hopper, & D. I. Rome (Eds.), *Mindfulness-oriented interventions for trauma: Integrating contemplative practices* (pp. 11–30). Guilford Press.

[CR17] Briere J, Hodges M, Godbout N (2010). Traumatic stress, affect dysregulation, and dysfunctional avoidance: A structural equation model. Journal of Traumatic Stress.

[CR18] Busby DM, Holman TB, Taniguchi N (2001). RELATE: Relationship evaluation of the individual, family, cultural, and couple contexts. Family Relations.

[CR19] Cloitre M, Stolbach BC, Herman JL, Kolk BVD, Pynoos R, Wang J, Petkova E (2009). A developmental approach to complex PTSD: Childhood and adult cumulative trauma as predictors of symptom complexity. Journal of Traumatic Stress.

[CR20] Colman RA, Widom CS (2004). Childhood abuse and neglect and adult intimate relationships: A prospective study. Child Abuse & Neglect.

[CR21] Dekeyser M, Raes F, Leijssen M, Leysen S, Dewulf D (2008). Mindfulness skills and interpersonal behaviour. Personality and Individual Differences.

[CR22] Déziel J, Godbout N, Hébert M (2017). Anxiety, dispositional mindfulness, and sexual desire in men consulting in clinical sexology: A mediational model. Journal of Sex & Marital Therapy.

[CR23] Dugal, C., Bigras, N., Godbout, N., & Bélanger, C. (2016). Childhood interpersonal trauma and its repercussions in adulthood: An analysis of psychological and interpersonal sequelae. In G. El-Baalbaki, & C. Fortin (Eds.), *A multidimensional approach to post-traumatic stress disorder: From theory to practice* (pp. 71–107). IntechOpen.

[CR24] Elices M, Pascual JC, Carmona C, Martín-Blanco A, Feliu-Soler A, Ruiz E, Gomà-i-Freixanet M, Pérez V, Soler J (2015). Exploring the relation between childhood trauma, temperamental traits and mindfulness in borderline personality disorder. BMC Psychiatry.

[CR25] Evans GW, Li D, Whipple SS (2013). Cumulative risk and child development. Psychological Bulletin.

[CR26] Faulkner RA, Davey M, Davey A (2005). Gender-related predictors of change in marital satisfaction and marital conflict. The American Journal of Family Therapy.

[CR27] Finkelhor D, Ormrod R, Turner HA (2007). Poly-victimization: A neglected component in child victimization. Child Abuse & Neglect.

[CR28] Finkelhor D, Ormrod RK, Turner HA (2009). Lifetime assessment of poly-victimization in a national sample of children and youth. Child Abuse & Neglect.

[CR29] Fisak B, Von Lehe AC (2012). The relation between the five facets of mindfulness and worry in a non-clinical sample. Mindfulness.

[CR30] Gambrel LE, Piercy FP (2015). Mindfulness-based relationship education for couples expecting their first child—Part 1: A randomized mixed-methods program evaluation. Journal of Marital and Family Therapy.

[CR31] Gambrel LE, Piercy FP (2015). Mindfulness-based relationship education for couples expecting their first child—Part 2: Phenomenological findings. Journal of Marital and Family Therapy.

[CR32] Godbout, N., Bigras, N., & Sabourin, S. (2017a).* Childhood cumulative trauma questionnaire* (CCTQ). Department of Psychology, University of Quebec at Montreal, Canada.

[CR33] Godbout N, Briere J, Sabourin S, Lussier Y (2014). Child sexual abuse and subsequent relational and personal functioning: The role of parental support. Child Abuse & Neglect.

[CR34] Godbout N, Daspe M-È, Lussier Y, Sabourin S, Dutton D, Hébert M (2017). Early exposure to violence, relationship violence, and relationship satisfaction in adolescents and emerging adults: The role of romantic attachment. Psychological Trauma Theory Research Practice and Policy.

[CR35] Godbout N, Lussier Y, Sabourin S (2006). Early abuse experiences and subsequent gender differences in couple adjustment. Violence and Victims.

[CR36] Godbout N, Sabourin S, Lussier Y (2007). The relation between sexual abuse undergone during childhood and male marital satisfaction. Canadian Journal of Behavioural Science.

[CR37] Graham JM, Liu YJ, Jeziorski JL (2006). The dyadic adjustment scale: A reliability generalization meta-analysis. Journal of Marriage and Family.

[CR38] Gu J, Strauss C, Crane C, Barnhofer T, Karl A, Cavanagh K, Kuyken W (2016). Examining the factor structure of the 39-item and 15-item versions of the five facet mindfulness questionnaire before and after mindfulness-based cognitive therapy for people with recurrent depression. Psychological Assessment.

[CR39] Hayes SC, Gifford EV (1997). The trouble with language: Experiential avoidance, rules, and the nature of verbal events. Psychological Science.

[CR40] Heeren A, Douilliez C, Peschard V, Debrauwere L, Philippot P (2011). Cross-cultural validity of the five facets mindfulness questionnaire: Adaptation and validation in a French-speaking sample. European Review of Applied Psychology.

[CR41] Hodges M, Godbout N, Briere J, Lanktree C, Gilbert A, Kletzka NT (2013). Cumulative trauma and symptom complexity in children: A path analysis. Child Abuse & Neglect.

[CR42] Holt-Lunstad J, Smith TB, Layton JB (2010). Social relationships and mortality risk: A meta-analytic review. PLoS Medicine.

[CR43] Hooper D, Coughlan J, Mullen MR (2008). Structural equation modelling: Guidelines for determining model fit. Electronic Journal of Business Research Methods.

[CR44] Hu LT, Bentler PM (1999). Cutoff criteria for fit indexes in covariance structure analysis: Conventional criteria versus new alternatives. Structural Equation Modeling: A Multidisciplinary Journal.

[CR45] Iani L, Lauriola M, Cafaro V, Didonna F (2017). Dimensions of mindfulness and their relations with psychological well-being and neuroticism. Mindfulness.

[CR46] Johns KN, Allen ES, Gordon KC (2015). The relationship between mindfulness and forgiveness of infidelity. Mindfulness.

[CR47] Jones SM, Hansen W (2015). The impact of mindfulness on supportive communication skills: Three exploratory studies. Mindfulness.

[CR48] Kabat-Zinn J (2003). Mindfulness-based interventions in context: Past, present, and future. Clinical Psychology: Science and Practice.

[CR49] Kappen G, Karremans JC, Burk WJ, Buyukcan-Tetik A (2018). On the association between mindfulness and romantic relationship catisfaction: The role of partner acceptance. Mindfulness.

[CR50] Karremans JC, Schellekens MPJ, Kappen G (2017). Bridging the sciences of mindfulness and romantic relationships: A theoretical model and research agenda. Personality and Social Psychology Review.

[CR51] Kelly A, Garland EL (2016). Trauma-informed mindfulness-based stress reduction for female survivors of interpersonal violence: Results from a stage I RCT. Journal of Clinical Psychology.

[CR52] Kline, R. B. (2015).* Principles and practice of structural equation modeling*. Guilford Publications.

[CR53] Kozlowski A (2013). Mindful mating: Exploring the connection between mindfulness and relationship satisfaction. Sexual and Relationship Therapy.

[CR54] Kratzer L, Heinz P, Pfitzer F, Padberg F, Jobst A, Schennach R (2018). Mindfulness and pathological dissociation fully mediate the association of childhood abuse and PTSD symptomatology. European Journal of Trauma & Dissociation.

[CR55] Lilja JL, Lundh L-G, Josefsson T, Falkenström F (2013). Observing as an essential facet of mindfulness: A comparison of FFMQ patterns in meditating and non-meditating individuals. Mindfulness.

[CR56] Linehan, M. M. (2014). *DBT skills training manual*. Guilford Publications.

[CR57] Masten AS, Wright MOD (1998). Cumulative risk and protection models of child maltreatment. Journal of Aggression, Maltreatment & Trauma.

[CR58] McCrory EJ, Viding E (2015). The theory of latent vulnerability: Reconceptualizing the link between childhood maltreatment and psychiatric disorder. Development and Psychopathology.

[CR59] Muthén, L. K., & Muthén, B. O. (2017). *Mplus user’s guide* (8th ed.). Muthén & Muthén.

[CR60] Nguyen TP, Karney BR, Bradbury TN (2017). Childhood abuse and later marital outcomes: Do partner characteristics moderate the association?. Journal of Family Psychology.

[CR61] Nitzan-Assayag Y, Aderka IM, Bernstein A (2015). Dispositional mindfulness in trauma recovery: Prospective relations and mediating mechanisms. Journal of Anxiety Disorders.

[CR62] Norman RE, Byambaa M, De R, Butchart A, Scott J, Vos T (2012). The long-term health consequences of child physical abuse, emotional abuse, and neglect: A systematic review and meta-analysis. PLoS Medicine.

[CR63] Papies EK, Pronk TM, Keesman M, Barsalou LW (2015). The benefits of simply observing: Mindful attention modulates the link between motivation and behavior. Journal of Personality and Social Psychology.

[CR64] Park T, Reilly-Spong M, Gross CR (2013). Mindfulness: A systematic review of instruments to measure an emergent patient-reported outcome (PRO). Quality of Life Research.

[CR65] Pepping, C. A., & Halford, W. K. (2016). Mindfulness and couple relationships. In Shonin, E., Van Gordon, W. & Griffiths, M. (Eds.), *Mindfulness and Buddhist-derived approaches in mental health and addiction* (pp. 391–411). Springer.

[CR66] Preacher KJ, Hayes AF (2008). Asymptotic and resampling strategies for assessing and comparing indirect effects in multiple mediator models. Behavior Research Methods.

[CR67] Rumstein-McKean O, Hunsley J (2001). Interpersonal and family functioning of female survivors of childhood sexual abuse. Clinical Psychology Review.

[CR68] Sabourin S, Valois P, Lussier Y (2005). Development and validation of a brief version of the dyadic adjustment scale with a nonparametric item analysis model. Psychological Assessment.

[CR69] Shapiro SL, Oman D, Thoresen CE, Plante TG, Flinders T (2008). Cultivating mindfulness: Effects on well-being. Journal of Clinical Psychology.

[CR70] Spanier GB (1976). Measuring dyadic adjustment: New scales for assessing the quality of marriage and similar dyads. Journal of Marriage and Family.

[CR71] Staples J, Rellini AH, Roberts SP (2012). Avoiding experiences: Sexual dysfunction in women with a history of sexual abuse in childhood and adolescence. Archives of Sexual Behavior.

[CR72] Statistique Canada (2020).* Low income cut-offs (LICOs) before and after tax by community size and family size, in constant dollars*. 10.25318/1110019501-eng.

[CR73] Suliman S, Mkabile SG, Fincham DS, Ahmed R, Stein DJ, Seedat S (2009). Cumulative effect of multiple trauma on symptoms of posttraumatic stress disorder, anxiety, and depression in adolescents. Comprehensive Psychiatry.

[CR74] Tavakolizadeh J, Nejatian M, Soori A (2015). The effectiveness of communication skills training on marital conflicts and its different aspects in women. Procedia - Social and Behavioral Sciences.

[CR75] Thompson BL, Waltz J (2010). Mindfulness and experiential avoidance as predictors of posttraumatic stress disorder avoidance symptom severity. Journal of Anxiety Disorders.

[CR76] Vaillant GE, Mukamal K (2001). Successful aging. American Journal of Psychiatry.

[CR77] Van Dam NT, van Vugt MK, Vago DR, Schmalzl L, Saron CD, Olendzki A, Meissner T, Lazar SW, Kerr CE, Gorchov J (2018). Mind the hype: A critical evaluation and prescriptive agenda for research on mindfulness and meditation. Perspectives on Psychological Science.

[CR78] Veehof MM, ten Klooster PM, Taal E, Westerhof GJ, Bohlmeijer ET (2011). Psychometric properties of the Dutch Five Facet Mindfulness Questionnaire (FFMQ) in patients with fibromyalgia. Clinical Rheumatology.

[CR79] Vujanovic AA, Youngwirth NE, Johnson KA, Zvolensky MJ (2009). Mindfulness-based acceptance and posttraumatic stress symptoms among trauma-exposed adults without axis I psychopathology. Journal of Anxiety Disorders.

[CR80] Whisman M (2006). Childhood trauma and marital outcomes in adulthood. Personal Relationships.

[CR81] Whisman M, du Pont A, Rhee SH, Spotts EL, Lichtenstein P, Ganiban JM, Reiss D, Neiderhiser JM (2018). A genetically informative analysis of the association between dyadic adjustment, depressive symptoms, and anxiety symptoms. Journal of Affective Disorders.

[CR82] Whisman M, Uebelacker LA (2006). Impairment and distress associated with relationship discord in a national sample of married or cohabiting adults. Journal of Family Psychology.

